# miR-98-5p Alleviated Epithelial-to-Mesenchymal Transition and Renal Fibrosis via Targeting Hmga2 in Diabetic Nephropathy

**DOI:** 10.1155/2019/4946181

**Published:** 2019-11-26

**Authors:** Yingchun Zhu, Jiang Xu, Wenxing Liang, Ji Li, Linhong Feng, PengXi Zheng, Tingting Ji, Shoujun Bai

**Affiliations:** ^1^Department of Nephrology, Qingpu Branch of Zhongshan Hospital Affiliated to Fudan University, 1158 Gongyuan East Road, Qingpu District, Shanghai 201700, China; ^2^Department of Rehabilitation, Huai'an Second People's Hospital, The Affiliated Hospital of Xuzhou Medical University, Huai'an, China

## Abstract

Recently, microRNAs have been recognized as crucial regulators of diabetic nephropathy (DN) development. Epithelial-to-mesenchymal transition (EMT) can play a significant role in tubulointerstitial fibrosis, and it is a hallmark of diabetic nephropathy progression. Nevertheless, the function of miR-98-5p in the modulation of EMT and renal fibrosis during DN remains barely investigated. Hence, identifying the mechanisms of miR-98-5p in regulating EMT and fibrosis is of huge significance. In our present research, decreased miR-98-5p was demonstrated in db/db mice and mice mesangial cells treated with the high dose of glucose. Meanwhile, activated EMT and increased fibrosis was accompanied with the decrease of miR-98-5p *in vitro* and *in vivo*. Additionally, to further find out the roles of miR-98-5p in DN development, overexpression of miR-98-5p was applied. Firstly, *in vivo* investigation exhibited that elevation of miR-98-5p restrained proteinuria, serum creatinine, BUN, the EMT process, and fibrosis. Furthermore, high glucose was able to promote mice mesangial cell proliferation, EMT process, and induced renal fibrosis, which could be prevented by overexpression of miR-98-5p. Moreover, high mobility group A (HMGA2) can exhibit an important role in diverse biological processes. Here, HMGA2 was investigated as a target of miR-98-5p currently. Luciferase reporter assay was conducted and the correlation of miR-98-5p and HMGA2 was validated. Moreover, it was displayed that HMGA2 was remarkably elevated in db/db mice and mice mesangial cells. Furthermore, miR-98-5p strongly depressed HMGA2 protein and mRNA levels in mice mesangial cells. Overall, these revealed miR-98-5p could suppress the EMT process and renal fibrosis through targeting HMGA2 in DN.

## 1. Introduction

DN is a common complication of diabetes, which can contribute to ESRD all over the world [1, 2]. Increasing evidences have demonstrated that inflammatory process, oxidative stress, and autophagy are responsible for the progression of DN [3–5]. As a frequent complication of diabetes, DN still cannot be treated effectively, and it is urgent to develop more effective methods to repress its progression [6].

As well known, EMT is a kind of biological course, and epithelial cells can transdifferentiate into mesenchymal cells in this process. This process is able to contribute a lot to pathological fibrosis and cancer development [7, 8]. As reported, in the period of EMT, epithelial cells will drop the apical-basal polarity and junctions, which leads to the phenotypes of mesenchymal cells. The mesenchymal cells have increased migratory and invasive ability, which results in the accumulation of ECM components [9]. For example, epithelial cells going through the EMT process are involved in kidney fibrosis [10]. Moreover, in human renal tissues, mesenchymal indicator‐positive TECs is related with the upregulated serum creatinine levels [11]. These studies have revealed a crucial role of EMT and renal fibrosis in DN pathogenesis.

MicroRNAs are small noncoding RNAs involved in multiple processes [12]. They can regulate protein expression via mRNA degradation or translational repression [13–15]. In recent studies, they have reported that increasing number of microRNAs can regulate the progression of DN [16]. For instance, miR-146a exerts an anti-inflammation role in DN pathogenesis [17]. miR-27a induces podocyte injury through activation of *β*-catenin in DN [18]. Besides these, via targeting PTEN and SMAD7, miR-21 promotes renal fibrosis in DN [19]. Up to now, the biological function of miR-98-5p in the EMT process and renal fibrosis of DN progression remains barely investigated. Hence, we concentrated on the mechanisms of miR-98-5p in DN development.

Currently, we hypothesized that miR-98-5p was involved in DN via the EMT process. In our present study, it was revealed miR-98-5p was a significant modulator of EMT and renal fibrosis in DN via targeting HMGA2. Enhancement of miR-98-5p attenuated mice mesangial cells proliferation and the progression of EMT and renal fibrosis.

## 2. Materials and Methods

### 2.1. Animals

Animal experiments were based on the standards of the NIH Instructions for the Care and Use of Laboratory Animals. The study was approved by the ethics committee of Qingpu Branch of Zhongshan Hospital affiliated to Fudan University. Male db/db mice with the background of C57BL/Ks and the control C57BL/Ks mice were obtained from Model Animal Research Center of Nanjing University (Nanjing, China). Mice were kept in the center with 12-hour light and 12-hour dark periods, and they were provided water and food without limitations. Db/db mice were divided into two groups (LV-NC and LV-miR-98-5p, *n*=8 for every group) at random. At the age of 12 weeks, the mice were injected with LV-miR-98-5p or LV-NC via the tail vein. Subsequently, anaesthetization was carried out by a xylazine-ketamine mixture intraperitoneal injection before all the mice were sacrificed at 24 weeks.

### 2.2. Blood and Urine Determination

Blood glucose was tested using the Glucose LiquiColor Test (Stanbio Laboratory, Boerne, TX, USA). 24-hour urine was obtained using the metabolic cage every four weeks. Serum creatinine, BUN level, and urinary creatinine level were examined on an AEROSET clinical chemistry system (Abbott Laboratories, Chicago, IL, USA). Urine albumin concentration was detected using the mice albumin ELISA kit (Bethyl Laboratories, Montgomery, TX, USA).

### 2.3. Cell Culture

Mouse mesangial cells and HEK-293T cells were purchased from the ATCC (Manassas, VA, USA). DMEM medium with 10% FBS (Invitrogen, Carlsbad, CA, USA), penicillin (100 U/ml), and streptomycin (100 *μ*g/ml) was employed to maintain all the cells. Cells were grown at a 5% CO2 atmosphere at 37°C.

### 2.4. CCK8 Assay

Cells were plated in a 96-well plate for a whole night. Then, cell viability was tested using the CCK-8 method (CCK8, Dojindo, Japan).

### 2.5. EdU Assay

EdU experiment was conducted using a Cell-Light EdU DNA Cell Proliferation Kit (RiboBio, Shanghai, PR, China). After treating with 50 mM EdU for two hours, 4% paraformaldehyde was used to fix the cells. Then, the cells were stained by Apollo Dye Solution with the nucleic acid stained by Hoechst-33342. Images were observed using an Olympus FSX100 microscope.

### 2.6. qRT-PCR

Total RNA was extracted by TRIzol Reagent (Invitrogen, Carlsbad, CA, USA). RNA was reverse transcribed using the first-strand cDNA synthesis kit (Thermo Scientific, Waltham, MA, USA). Detection of the expression of miR-98-5p and mRNA expression of E-cadherin, N-cadherin, HMGA2, TGF-*β*1, COL4A1, and qPCR was carried out using a SYBR Premix Ex Taq II (TaKaRaBio Technology, Dalian, China) on the 7900HT Fast Real-Time PCR system (Applied Biosystems, Foster City, CA, USA). Primers were synthesized by GenePharma (Shanghai, China). The primers used are listed in Table 1. U6 RNA was employed as an internal microRNA control. GAPDH acted as an internal mRNA control. Fold change was calculated using 2^−ΔΔCt^.

### 2.7. Western Blot

Cells were harvested, and the lysis buffer was used to extract the cell proteins. Protein extracts were boiled, and cell extracts were isolated on 10% SDS-PAGE gels. Then, the protein bands were transferred onto PVDF membranes. The primary antibodies E-cadherin, N-cadherin, HMGA2, TGF-*β*1, COL4A1, and GAPDH were incubated with the membranes for a whole night at 4°C. The next day, secondary antibodies linked by HRP were used. The immunoreactive bands were exposed using ECL-PLUS/Kit (GE Healthcare, Piscataway, NJ, USA).

### 2.8. Dual Luciferase Assay

The binding sites between HMGA2 and miR-98-5p were predicted by TargetScan (http://www.targetscan.org/vert_71/). The 3ʹ-untranslated region (UTR) of HMGA2 was amplified from cDNA of HEK-293T cells and inserted into pMIR (Promega Corporation, Madison, WI, USA). pMIR-HMGA2 3ʹ-UTR or pMIR-HMGA2 3ʹ-UTR mutant was transfected into HEK-293T cells using Lipofectamine 2000 (Invitrogen, Carlsbad, CA, USA). After 24 hours, the activity of luciferase and *Renilla* activity were detected using a Dual Luciferase Reporter Assay Kit (Promega, Madison, WI, USA).

### 2.9. Statistical Analysis

Data were manifested as mean ± SD and analyzed by SPSS 19.0 software (SPSS, Inc., Chicago, IL, USA). The Student's *t*-test and ANOVA were carried out among different groups. Differences with *P* < 0.05 were considered to be significant.

## 3. Results

### 3.1. miR-98-5p was Downregulated in DN

**Figure 1:**
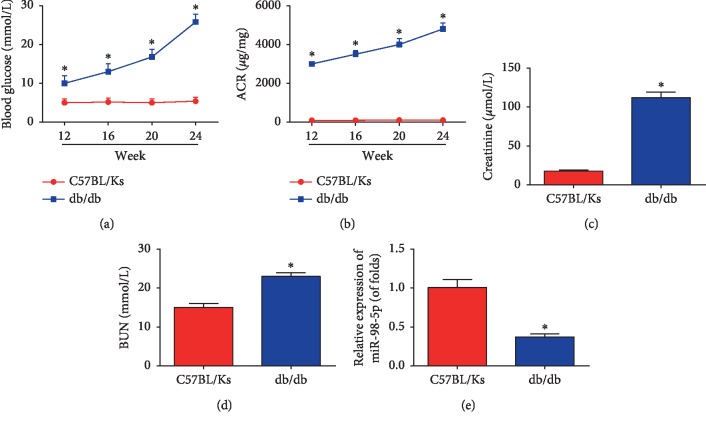
miR-98-5p was decreased in db/db mice. (a) Blood glucose was determined every 4 weeks since the age of 12 weeks. (b) Urine ACR was detected every 4 weeks since the age of 12 weeks. (c) Serum creatinine was examined at the age of 24 weeks. (d) BUN was determined at the age of 24 weeks. (e) miR-98-5p expression in the renal cortex was measured by real-time PCR. *n*=8 for each group. Data are representative of three experiments. Error bars stand for the mean ± SD of at least triplicate experiments. ^*∗*^*P* < 0.05.

Firstly, to investigate the role of miR-98-5p in DN development, db/db mice with C57BL/Ks background were employed in our study. During the period, we observed that the blood glucose and the urine ACR in db/db mice were increased from 12 weeks progressively (Figures 1(a) and 1(b)). Additionally, at 24 weeks, the serum creatinine and BUN were examined and as exhibited, they were remarkably upregulated in db/db mice (Figures 1(c) and 1(d), *n*=8 for each group). Furthermore, as shown in Figure 1(e), in the kidney tissues of db/db mice, miR-98-5p was greatly decreased (*n*=8 for each group).

### 3.2. miR-98-5p Reduced the Renal Dysfunction of db/db Mice

**Figure 2:**
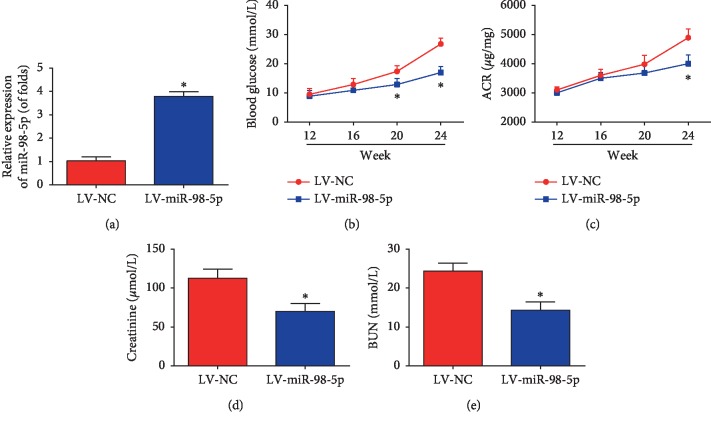
Overexpression of miR-98-5p attenuated renal dysfunction in db/db mice. (a) miR-98-5p expression in the renal cortex was tested using real-time PCR. (b) Blood glucose was determined every 4 weeks. (c) Urine ACR was detected every 4 weeks. (d) Serum creatinine was examined at the age of 24 weeks. (e) BUN was determined at the age of 24 weeks. *n*=8 for each group. Data are representative of three experiments. Error bars stand for the mean ± SD of at least triplicate experiments. ^*∗*^*P* < 0.05.

Then, to find out whether miR-98-5p can modulate renal dysfunction of db/db mice, the level of miR-98-5p in db/db mice was modulated by the lentivirus system. By LV-miR-98-5p delivery, as exhibited, miR-98-5p was efficiently increased in the renal cortex after 3 months (Figure 2(a), *n*=8 for each group). In Figure 2(b), increase of miR-98-5p reduced hyperglycemia development. Besides, LV-miR-98-5p treatment attenuated the urinary protein excretion compared with the control mice (Figure 2(c)). Additionally, overexpression of miR-98-5p repressed creatinine of the serum and BUN level (Figures 2(d) and 2(e), *n*=8 for each group).

### 3.3. EMT and Renal Fibrosis Was Induced in db/db Mice

**Figure 3:**
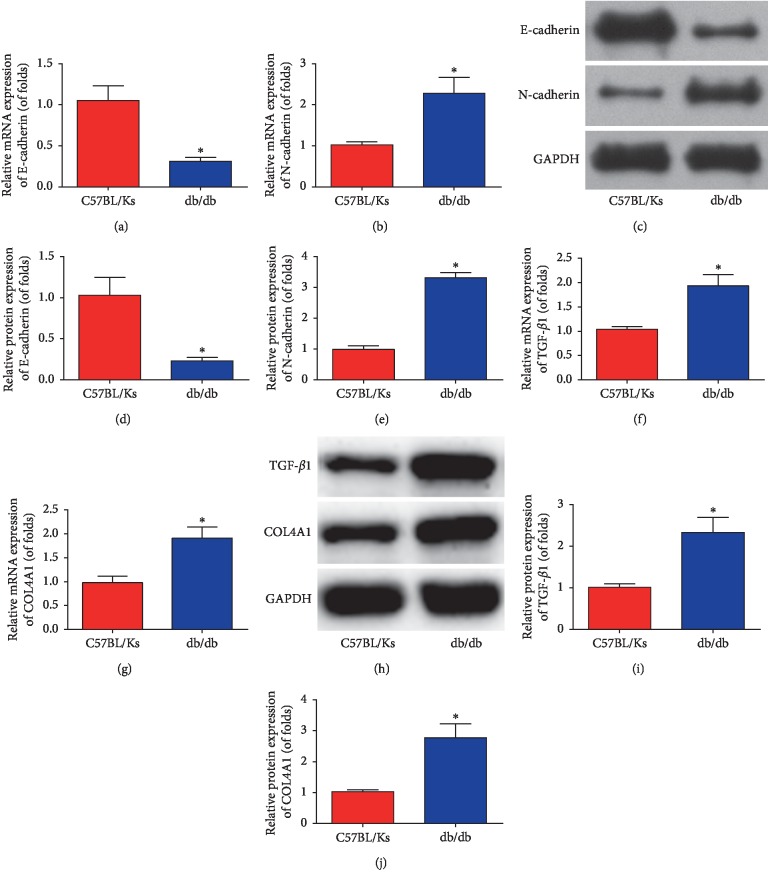
EMT and renal fibrosis was induced in db/db mice. mRNA expression of E-cadherin (a) and N-cadherin (b). The western blot images of E-cadherin and N-cadherin (c). Quantification of the western blot images of E-cadherin and N-cadherin (d and e). mRNA expression of TGF-*β*1 (f) and COL4A1 (g). The western blot images of TGF-*β*1 and COL4A1 (h). Quantification of the western blot images of TGF-*β*1 (i) and COL4A1 (j). *n*=8 for each group. Data are representative of three experiments. Error bars stand for the mean ± SD of at least triplicate experiments. ^*∗*^*P* < 0.05.

Next, the expression of EMT biomarkers (E-cadherin and N-cadherin) was determined using qRT-PCR and western blot assays in the renal cortex. The results in Figures 3(a) and 3(b) showed that E-cadherin mRNA expression was remarkably reduced in db/db mice, while N-cadherin was enhanced (*n*=8 for each group). Consistent results were observed in western blot experiments. Figures 3(c)–3(e) exhibit that EMT was obviously triggered in db/db mice (*n*=8 for each group). Meanwhile, renal fibrosis biomarkers (TGF-*β*1 and COL4A1) were tested using qRT-PCR and western blots. The results in Figures 3(f) and 3(g) indicated that TGF-*β*1 and COL4A1 mRNA expression were obviously elevated in db/db mice (*n*=8 for each group). In addition, TGF-*β*1 and COL4A1 protein expression were also increased in db/db mice (Figures 3(h)–3(j), *n*=8 for each group).

### 3.4. EMT and Renal Fibrosis Were Repressed by miR-98-5p in db/db Mice

**Figure 4:**
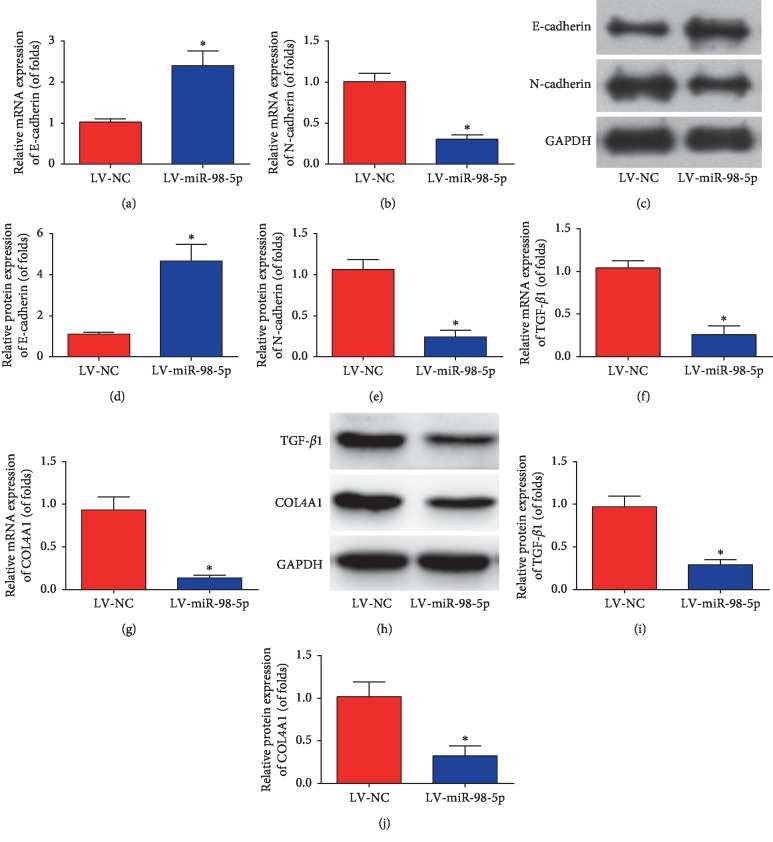
EMT and renal fibrosis were restrained by overexpression of miR-98-5p in db/db mice. mRNA expression of E-cadherin (a) and N-cadherin (b). Western blot images of E-cadherin and N-cadherin (c). Quantification of the western blot images of E-cadherin and N-cadherin (d and e). mRNA expression of TGF-*β*1 (f) and COL4A1 (g). The western blot images of TGF-*β*1 and COL4A1 (h). Quantification of the western blot images of TGF-*β*1 (i) and COL4A1 (j). *n*=8 for each group. Data are representative of three experiments. Error bars stand for the mean ± SD of at least triplicate experiments. ^*∗*^*P* < 0.05.

Moreover, after miR-98-5p was greatly overexpressed, shown in Figures 4(a) and 4(b), E-cadherin mRNA level was obviously increased with a decrease of N-cadherin mRNA expression (*n*=8 for each group). Apart from these, western blot assays shown in Figures 4(c)–4(e) manifested that E-cadherin protein was induced and N-cadherin was reduced by miR-98-5p upregulation in db/db mice (*n*=8 for each group). Meanwhile, renal fibrosis biomarkers (TGF-*β*1 and COL4A1) were tested using qRT-PCR and western blot assays. Furthermore, we found that TGF-*β*1 and COL4A1 mRNA expression were obviously repressed by overexpression of miR-98-5p *in vivo* (Figures 4(f) and 4(g), *n*=8 for each group). Consistently, TGF-*β*1 and COL4A1 protein expression were restrained by miR-98-5p in db/db mice (Figures 4(h)–4(j), *n*=8 for each group).

### 3.5. miR-98-5p Suppressed Mouse Mesangial Cell Proliferation, EMT, and Renal Fibrosis

**Figure 5:**
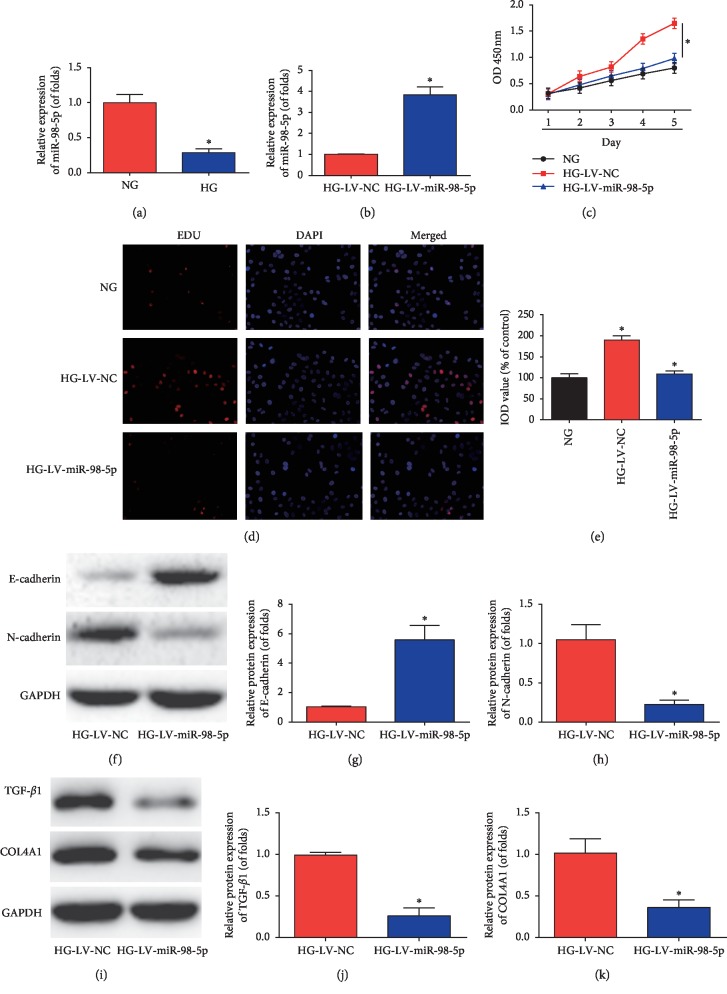
Overexpression of miR-98-5p alleviated high glucose-induced cell proliferation, EMT, and renal fibrosis in cultured mice mesangial cells. (a) Real-time qPCR analysis showing miR-98-5p expression in mice mesangial cells treated with high glucose (HG, 25 mM) as compared with the cells treated with normal glucose (NG, 5.6 mM). (b) Real-time qPCR analysis showing miR-98-5p expression in mice mesangial cells infected with LV-miR-98. (c) Result of CCK-8 assay in mouse mesangial cells. (d and e) Result of EDU assay in mouse mesangial cells. Western blot images of E-cadherin and N-cadherin (f). Quantification of the western blot images of E-cadherin and N-cadherin (g and h). The western blot images of TGF-*β*1 and COL4A1 (i). Quantification of the western blot images of TGF-*β*1 (j) and COL4A1 (k). *n*=8 for each group. Data are representative of three experiments. Error bars stand for the mean ± SD of at least triplicate experiments. ^*∗*^*P* < 0.05.

Furthermore, as indicated in Figure 5(a), miR-98-5p expression was upregulated in mouse mesangial cells treated with HG (25 mM glucose). To study whether miR-98-5p regulated mouse mesangial cell proliferation, LV-miR-98-5p was infected into mouse mesangial cells and it was successfully increased (Figure 5(b)). CCK-8 assay was conducted, and as displayed in Figure 5(c), LV-miR-98-5p greatly repressed cell growth. Additionally, EDU assay (Figures 5(d) and 5(e)) implied that cell proliferation was triggered by high glucose and miR-98-5p reversed this process. Besides these, results of western blot assays shown in Figures 5(f)–5(h) suggested that E-cadherin protein was increased and N-cadherin was inhibited by miR-98-5p *in vitro*. Additionally, in Figures 5(i)–5(k), western blot data revealed that TGF-*β*1 and COL4A1 protein expression were retarded by miR-98-5p.

### 3.6. miR-98-5p Targeted HMGA2

**Figure 6:**
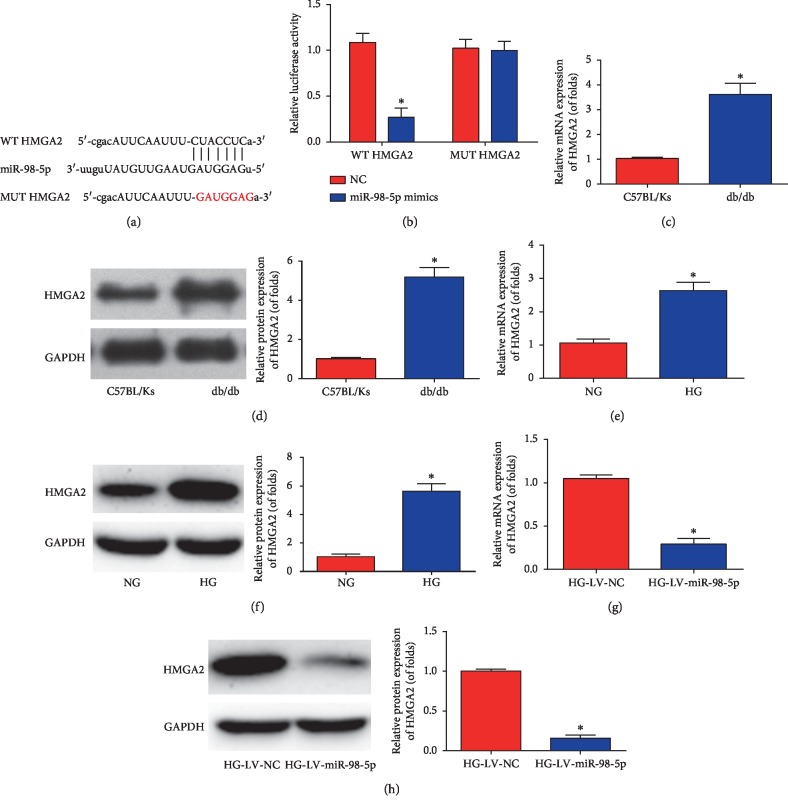
HMGA2 was a direct target of miR-98-5p. (a) The luciferase reporter constructs containing the wild type (WT-HMGA2) or mutant HMGA2 (MUT-HMGA2) sequence. (b) WT-HMGA2 or MUT-HMGA2 was cotransfected into HEK-293T cells with miR-98-5p mimics or their corresponding negative controls. (c) mRNA expression of HMGA2 in db/db mice. (d) Protein expression of HMGA2 in db/db mice. (e) mRNA expression of HMGA2 in mouse mesangial cells. (f) Protein expression of HMGA2 in mouse mesangial cells. (g) mRNA expression of HMGA2 in mouse mesangial cells transfected with LV-miR-98-5p. (h) Protein expression of HMGA2 in mouse mesangial cells transfected with LV-miR-98-5p. Data are representative of three experiments. Error bars stand for the mean ± SD of at least triplicate experiments. ^*∗*^*P* < 0.05.

Subsequently, HMGA2 was searched as the target of miR-98-5p. Luciferase reporter plasmids of WT-HMGA2 and MUT-HMGA2 binding sites were manifested in Figure 6(a). Cotransfection of WT-HMGA2 with miR-98-5p mimics suppressed the reporter activity in HEK-293T cells (Figure 6(b)). Figures 6(c) and 6(d) show that HMGA2 was strongly induced in the kidney tissues of db/db mice. Then, we found HMGA2 expression was elevated in mouse mesangial cells indicating high glucose, as shown in Figures 6(e) and 6(f). HMGA2 was strongly inhibited by miR-98-5p overexpression in mouse mesangial cells, as shown in Figures 6(g) and 6(h).

## 4. Discussion

MicroRNAs can participate in various cardiovascular processes, which are closely correlated with numerous cardiovascular diseases, such as coronary heart disease, hypertension, and myocardial infarction [20]. Accumulating studies have indicated that microRNAs are dysregulated in DN progression [21–23]. Here, miR-98-5p/HMGA2 axis was identified as a novel mechanism in development of DN. As exhibited, miR-98-5p was decreased in db/db mice and mice mesangial cells indicated with high glucose, whereas HMGA2 was greatly increased. Moreover, mouse mesangial cell proliferation and EMT process were strongly prevented by miR-98-5p overexpression.

miR-98 is a member of a highly conserved small RNA family. It acts as a crucial tumor suppressor in several tumors. For example, miR-98 can inhibit HCC progression via EZH2 and inactivating Wnt/*β*-catenin [24]. By targeting ITGB3, miR-98 inhibits the progression of NSCLC [25]. Additionally, another study indicates that high glucose concentration can trigger proliferation of endothelial cells by regulating miR-98 [26]. Here, we studied that miR-98-5p was decreased in db/db mice and mice mesangial cells, which are treated with high glucose. In addition, we observed that overexpression of miR-98-5p reduced hyperglycemia development and the renal dysfunction of db/db mice. The improvement of kidney functions might be due to the improvement of hyperglycemia. In our future study, we would like to investigate this.

EMT plays a crucial role in the progression of DN [27]. For instance, miR-30c can protect DN by suppressing EMT *in vivo* [28]. miR-23b can function as an EMT suppressor in DN through inactivating PI3K-AKT pathway activation [10]. In addition, miR-130b can protect renal tubulointerstitial fibrosis via repressing EMT in DN [29]. Furthermore, previous research studies have indicated that miR-98 can inhibit the EMT process in several cancers. miR-98 can repress TWIST expression to prevent the progression of NSCLC [30]. MiR-98 prevents the invasion capacity and EMT of HCC [31]. Here, in our study, it was observed that EMT was greatly triggered in db/db mice and overexpression of miR-98-5p was able to restrain EMT process in DN. As exhibited, E-cadherin expression was induced by LV-miR-98-5p, whereas N-cadherin was suppressed by overexpression of miR-98-5p. In addition, we observed that renal fibrosis biomarkers TGF-*β*1 and COL4A1 were greatly induced in db/db mice. Overexpression of miR-98-5p remarkably inhibited TGF-*β*1 and COL4A1 *in vitro* and *in vivo*.

HMGA2 has been documented to regulate adipogenesis and mesenchymal differentiation and promote benign mesenchymal tumors [32, 33]. Recent studies have demonstrated that HMGA2 exerts a critical role in DN progression. Let-7d can prevent the EMT process triggered by TGF-*β*1 and renal fibrogenesis via regulating HMGA2 expression [34]. Meanwhile, loss of HMGA2 weakens EMT in tubular epithelial cells [35]. Common variation of HMGA2 gene can greatly enhance nephropathy of type 2 diabetic patients [36]. In our research, it was displayed that HMGA2 was a target of miR-98-5p. We proved that HMGA2 was elevated in DN models. Upregulation of miR-98-5p repressed HMGA2 levels in mice mesangial cells.

## 5. Conclusions

In conclusion, a novel role of miR-98-5p/HMGA2 axis was implied in DN progression in our research. miR-98-5p might act as a meaningful biomarker for DN.

## Figures and Tables

**Table 1:** Primers used for real-time PCR.

Genes	Forward (5ʹ–3ʹ)	Reverse (5ʹ–3ʹ)
GAPDH	CAAGGTCATCCATGACAACTTTG	GTCCACCACCCTGTTGCTGTAG
miR-98-5p	ATCCAGTGCGTGTCGTG	TGCTTGAGGTAGTAAGTTG
U6	CTCGCTTCGGCAGCACA	AACGCTTCACGAATTTGCGT
E-cadherin	CAATCTCAAGCTCATGG	CCATTCGTTCAAGTAGTC
N-cadherin	GTGCATGAAGGACAGCCTCT	CCACCTTAAAATCTGCAGGC
HMGA2	TGGGAGGAGCGAAATCTAA	GGTGAACTCAAGCCGAAG
TGF-*β*1	TATTGAGCACCTTGGGCACT	ACCTCTCTGGGCTTGTTTCC
COL4A1	CTCTGGCTGTGGCAAATGTG	CCTCAGGTCCTTGCATTCCA

## Data Availability

The data used to support the findings of this study are available from the corresponding author upon request.
